# Transcranial ultrasound stimulation ameliorates dextran sulphate sodium-induced colitis and behavioural disorders by suppressing the inflammatory response in the brain

**DOI:** 10.1093/braincomms/fcaf119

**Published:** 2025-03-21

**Authors:** Yu-Chen Lin, Yi-Ju Pan, Shu-Ming Chang, Feng-Yi Yang

**Affiliations:** Department of Biomedical Imaging and Radiological Sciences, National Yang Ming Chiao Tung University, Taipei 11221, Taiwan; Department of Psychiatry, Far Eastern Memorial Hospital, New Taipei City 220, Taiwan; Department of Chemical Engineering and Materials Science, Yuan Ze University, Taoyuan City 320315, Taiwan; Department of Biomedical Imaging and Radiological Sciences, National Yang Ming Chiao Tung University, Taipei 11221, Taiwan; Department of Biomedical Imaging and Radiological Sciences, National Yang Ming Chiao Tung University, Taipei 11221, Taiwan

**Keywords:** ultrasound, neuroinflammation, inflammatory bowel disease, microbiota, intestinal inflammation

## Abstract

Inflammatory bowel disease (IBD) is associated with neuroinflammation, which may contribute to an increased risk of neurodegenerative disorders. This research investigated the potential of transcranial low-intensity pulsed ultrasound (LIPUS) to mitigate colonic inflammation induced by dextran sulphate sodium (DSS), focusing on its effects via the brain–gut axis. Colitis and neuroinflammation were induced in mice by administering 3% (wt/vol) DSS for 7 days. Subsequently, the brain was subjected to LIPUS stimulation at intensities of 0.5 or 1.0 W/cm² for 3 days. Biological samples were analyzed using real-time polymerase chain reaction, western blot, enzyme-linked immunosorbent assay, and histological observation. Behavioural dysfunctions were assessed using the open field test, novel object recognition task, and Y-maze test. The alteration in gut microbiota composition was assessed through 16S rRNA sequencing. LIPUS therapy notably alleviated colitis symptoms and suppressed inflammation in both the colon and hippocampus of DSS-exposed mice. Compared with the group treated only with DSS, the LIPUS treatment showed decreased crypt destruction and partial epithelial barrier preservation. Moreover, LIPUS preserved intestinal barrier function by upregulating the levels of occludin and zonula occludens, decreasing the levels of lipopolysaccharide (LPS) and LPS-binding protein in serum, and ameliorating behavioural disorders. Further analysis indicated that LIPUS did not significantly transform the composition of the intestinal microbiota, but the microbial community showed some differences from the community in the DSS-only treatment group. This study demonstrates that transcranial LIPUS stimulation could be a novel therapeutic strategy for IBD and neuroinflammation via regulation of inflammatory interactions across brain–gut axis.

## Teaser

Low-intensity pulsed ultrasound alleviated dextran sulphate sodium-induced colonic and hippocampal inflammation through transcranial stimulation of mice. Transcranial LIPUS stimulation could be a novel therapeutic strategy for inflammatory bowel disease and neuroinflammation via regulation of inflammatory interactions across brain–gut axis signaling.

## Graphical Abstract

**Graphical Abstract:**
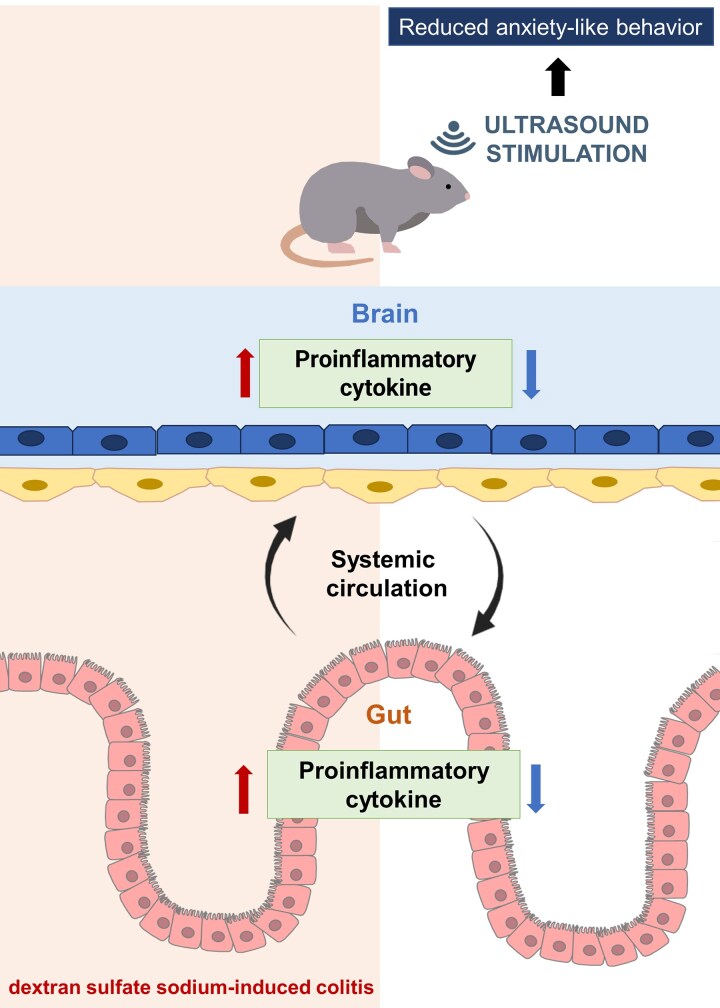

## Introduction

Two major inflammatory bowel diseases (IBDs) of the intestinal tract, Crohn’s disease and ulcerative colitis, are characterized by chronic inflammation and abdominal pain.^1,2^ Intestinal inflammation can cause central nervous system (CNS) inflammation, which potentially becomes one of the major pathogeneses of brain disorders.^3^ IBD is increasingly linked to neuroinflammation and behavioural changes in patients. However, the mechanisms underlying the communication between IBD and CNS are unknown. A number of clinical studies have shown that many IBD patients develop a variety of complications, including depressive symptoms and cognitive dysfunction.^4,5^ Therefore, it is of great importance to develop effective treatments for patients with IBD accompanied by neuroinflammation.

The brain–gut axis represents a complex bidirectional communication system comprising multiple interconnections between the CNS, the autonomic nervous system (ANS), and the enteric nervous system (ENS).^6,7^ Combined with activities in the ENS and modulation by the CNS, the ANS can induce CNS-modulated changes in the gut through top-down effects.^8^ Previous studies have proposed that psychological factors may influence IBD activity.^9-11^ Hormonal and neural lines of communication combine to allow the brain to influence intestinal functional activities, such as intestinal fluid handling, secretion of acid, and mucosal immune response, all controlled by the ANS.^12,13^ On the other hand, intestinal functional cells are modulated by the gut microbiota, whose contributing role in gut–brain reciprocal communications has recently been found.^14^ A number of reports have suggested that intestinal inflammation disturbs the gut microbiota composition and increases gut microbiota endotoxin levels.^15^ The connection between the gut and the brain may operate via the microbiota–gut–brain axis.^16^

The dextran sulphate sodium (DSS)-induced colitis model is widely used.^17^ DSS-induced intestinal inflammation triggers microglia activation as well as neuroinflammation.^18,19^ Furthermore, the inflammation between the brain and gut can exacerbate intestinal damage.^20^ Circulating proinflammatory factors can compromise epithelial tight junction integrity and aggravate neuroinflammation by promoting the secretion of proinflammatory mediators, including interleukin-1β (IL-1β), interleukin-6 (IL-6), and tumor necrosis factor-α (TNF-α),^21^ The increased intestinal permeability in patients with IBD facilitates the transit of several substances, such as bacteria-derived lipopolysaccharide (LPS), into the circulation, thus providing a pathway for proinflammatory cytokines to reach the brain and disrupt the blood–brain barrier (BBB).^22^ Therefore, the level of the LPS-binding protein (LBP) is associated with the inflammatory response, and the integrity of the intestinal barrier can be evaluated by the levels of LBP in serum.^23^

Low-intensity pulsed ultrasound (LIPUS) has significant anti-inflammatory properties; it decreases the levels of proinflammatory cytokines and increases the levels of epithelial tight junction proteins.^24,25^ In addition, LIPUS treatment decreases colitis severity by activating the splenic nerve and modulating the cholinergic anti-inflammatory pathway.^26^ However, whether transcranial stimulation by LIPUS can attenuate colonic inflammation is unclear. The purpose of this study was to explore the effects of transcranial LIPUS stimulation on DSS-induced colonic inflammation via inhibition of neuroinflammation. An acute colitis mouse model was developed with DSS, which could not cross the BBB and was suitable for exploring the direction of the inflammatory response from the gut to the brain.^27^ Our data showed that DSS-induced intestinal inflammation triggered systemic inflammation and neuroinflammation. We demonstrated that transcranial LIPUS stimulation can simultaneously alleviate DSS-induced neuroinflammation and colonic inflammation.

## Materials and methods

### Ultrasound system setup

The ultrasound system was similar to our previous study.^28^ Briefly, LIPUS was generated with an ultrasound apparatus (ME740, Mettler Electronics, Anaheim, CA) equipped with a 1-MHz plane transducer. The ultrasound settings comprised a 20% duty cycle, and a repetition frequency of 100 Hz. The spatial average intensity applied via the transducer was adjusted to either 0.5 W/cm² or 1.0 W/cm². Each sonication session lasted 5 min, with a 5-min interval between sessions, for a total stimulation duration of 15 min per day.

### Colitis model induction and experimental design

Eight-week-old C57BL/6J male mice (BioLASCO Co., Ltd., Yilan City, Taiwan) weighing 22–25 g were used in this study. The mice were housed under a 12 h/12 h light/dark cycle and given food ad libitum. Environmental conditions were standardized through consistent cage cleaning schedules, uniform bedding. This research received approval from the institutional Animal Care and Use Committee at National Yang Ming Chiao Tung University. Experimental mice were given drinking water containing 3% (wt/vol) DSS. The study utilized a randomized design to allocate mice into four distinct groups: Sham, DSS, DSS combined with LIPUS at 0.5 W/cm², and DSS combined with LIPUS at 1.0 W/cm² from Days 4 to 7. Mice were clinically evaluated for weight loss, stool consistency, and fecal bleeding, totalizing a disease activity index (DAI) (refer to Supplementary Table 1 for specific criteria).

### Quantitative real-time PCR

Total RNA was isolated from brain and colonic tissues using the RNeasy Mini Kit (QIAGEN, Germany) in accordance with the manufacturer's instructions. RNA concentration and purity were assessed using a NanoDrop ND-1000 spectrophotometer (Thermo Fisher Scientific, USA). Complementary DNA was synthesized using the ToolsQuant II Fast RT Kit (BioTools, Taiwan) according to the manufacturer’s guidelines. Quantitative real-time PCR (RT-PCR) was conducted with the SYBR (ABI Prism™, UK) in StepOnePlus™ (Applied Biosystems, USA).

### Western blot

Whole protein extracts were prepared from colonic tissue, and the total protein content was quantified. Proteins (30 μg per sample) were separated by electrophoresis and transferred onto PVDF membranes. Primary rabbit antibodies target inducible nitric oxide synthase (iNOS), occludin, and zonula occludens-1 (ZO-1). The specific bands were visualized using an ECL detection kit (Bio-Rad, CA, USA).

### Enzyme-linked immunosorbent assay

Serum levels of LBP, LPS and IL-6 were measured using enzyme-linked immunosorbent assay (ELISA) kits. The assays were performed in accordance with the manufacturers’ instructions.

### Colon length and histological assessment

The colon's length, measured from the ileocecal junction to the anus, was determined at the time of sacrifice, conducted 4 h following the terminal LIPUS stimulation. The distal sections of the colon were harvested, and a longitudinal slice was processed for analysis. Morphological alterations were evaluated through Hematoxylin and eosin (H&E) staining. The sections were imaged under a light microscope (Nikon E100, Japan) at 100× magnification. Colonic scoring was performed based on previously established criteria (Supplementary Table 2).^29^

### Assessment of behaviour

Three behavioural tests employed in this study are consistent with those used in our previous work.^30^ Briefly, the open field test (OFT) is utilized to assess behaviours indicative of depression and anxiety, as described in previous studies.^31^ The novel object recognition test (NORT) was employed to evaluate recognition memory. Following a 1-h habituation period in individual cages, mice underwent a training phase followed by a testing phase. The results were presented as a ratio of exploration. Mice with intact memory were expected to prefer the novel object, indicated by an exploration ratio exceeding 50%.^32^ The Y-maze paradigm was employed to evaluate spatial recognition memory. The proportion of time spent in the novel arm was determined by dividing the time spent exploring the novel arm by the total exploration time.^33^

### 16S rRNA sequencing analysis

The gut microbiota analysis followed a similar approach to that of our earlier research. In brief, total genomic DNA from feces was extracted from samples using the CatchGene™ Stool DNA Kit. The full-length 16S rRNA gene (V1–V9 regions) was amplified using primers with barcodes designed specifically for the 16S gene. The resulting sequences were clustered into operational taxonomic units with a 97% similarity threshold. Beta diversity analysis was conducted to evaluate variations in species diversity between samples and visualized through principal coordinate analysis (PCoA).

### Statistical analysis

All results are presented as mean values ± standard error of the mean (SEM). The Kolmogorov-Smirnov test was used to evaluate whether the data followed a normal distribution. With the exception of the 16S rRNA sequencing data, all analyses were conducted using one-way ANOVA, followed by Tukey’s *post hoc* test for group comparisons. The level of statistical significance was set at *P* < 0.05.

## Results

### Effects of DSS dose on mouse model with induced colitis

**Figure 1:**
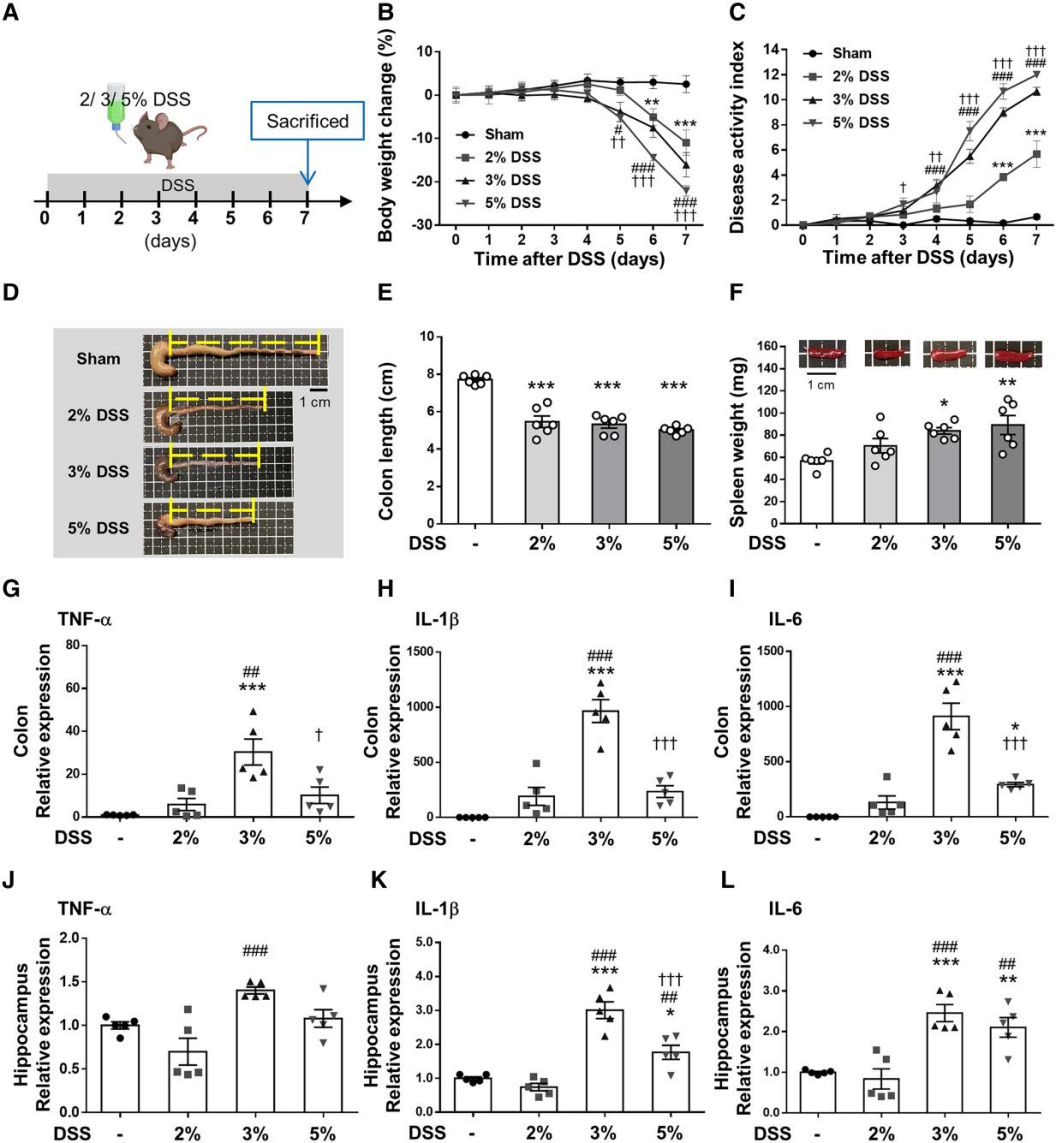
**DSS at different concentrations induces acute colitis and neuroinflammation in mice.** (**A**) Experimental design of the acute colitis animal model. Mice were treated with three doses of DSS dissolved in drinking water for 7 days and mice were sacrificed on Day 7. (**B**) Body weight changes are shown relative to Day 0 when weights were measured. (**C**) The 5, 3 and 2% DSS groups began to show a significant decrease in the DAI score compared with the Sham group starting from Days 3, 4 to 6, respectively (**B** and **C**, *n* = 6 for four groups). (**D**) Representative photographs of shortened colon. (**E**) Shrinkage of the colon in all three dose group. (**F**) Change in spleen weight in all three dose groups (**E** and **F**, *n* = 6 for four groups). RT-PCR analysis of the proinflammatory mediators (**G**) TNF-α, (**H**) IL-1β and (**I**) IL-6 in distal colon tissue. (**J**) TNF-α, (**K**) IL-1β and (**L**) IL-6 mRNA in the hippocampus was quantified with RT-PCR (G-L, *n* = 5 for four groups). In (**B**) and (**C**) *^, #^ and ^†^ denote a significant difference between the Sham group and the 2% DSS group, 3% DSS group and 5% DSS group, respectively. In (**E**)–(**L**), *^, #^ and ^†^ denote a significant difference from the Sham group, 2% DSS group and 3% DSS group, respectively (*^,#,†^, *P* < 0.05; **^,##,††^, *P* < 0.01; ***^,###,†††^, *P* < 0.001). All data are shown as mean ± SEM. Dots represent the number of samples (studied animals). One-way ANOVA followed by Tukey’s *post hoc* test. DAI, disease activity index; DSS, dextran sulphate sodium; IL-1β, interleukin-1β; IL-6, interleukin-6; TNF-α, tumor necrosis factor-α.

Three concentrations (2, 3 and 5%) of DSS were evaluated to find the optimum dose for this study (Fig. 1A). DSS administration for 7 days was associated with significant changes in body weight for all three doses compared with the Sham group (all *P* < 0.001; Fig. 1B). The DAI indicated the degree of severity of colitis during DSS administration in all three dose groups (Fig. 1C). The 5, 3 and 2% DSS groups began to show significant clinical signs of colitis compared with the Sham group starting from Day 3 (*P* < 0.05), Day 4 (*P* < 0.001), and Day 6 (*P* < 0.001), respectively. On Day 7, macroscopic observation of colons from the DSS groups showed colonic shortening compared with the Sham group (all *P* < 0.001; Fig. 1D and E). Furthermore, spleen weight was higher in the DSS group than in the Sham group, especially in the 3% DSS and 5% DSS groups (both *P* < 0.05; Fig. 1F).

To analyze the effects of DSS on inflammatory cytokine production in acute colitis and neuroinflammation, the proinflammatory cytokines, TNF-α, IL-1β and IL-6, in the colon and hippocampus were profiled. TNF-α, IL-1β and IL-6 were low in the sham-operated colons, but no significant difference was observed between the 2% DSS group and the Sham group. All proinflammatory cytokines were significantly induced in the 3% DSS and 5% DSS groups, especially in the 3% DSS group (all *P* < 0.05; Fig. 1G–I). Similarly, mRNA expression of TNF-α, IL-1β and IL-6 was significantly higher in the hippocampus of mice administered with DSS at the 3% dose (all *P* < 0.001; Fig. 1J–L). Based on the above results, the mice exposed to 3% DSS for 7 days were chosen to develop acute colitis and neuroinflammation in subsequent experiments.

### LIPUS brain stimulation attenuates DSS-induced colitis in mice

**Figure 2:**
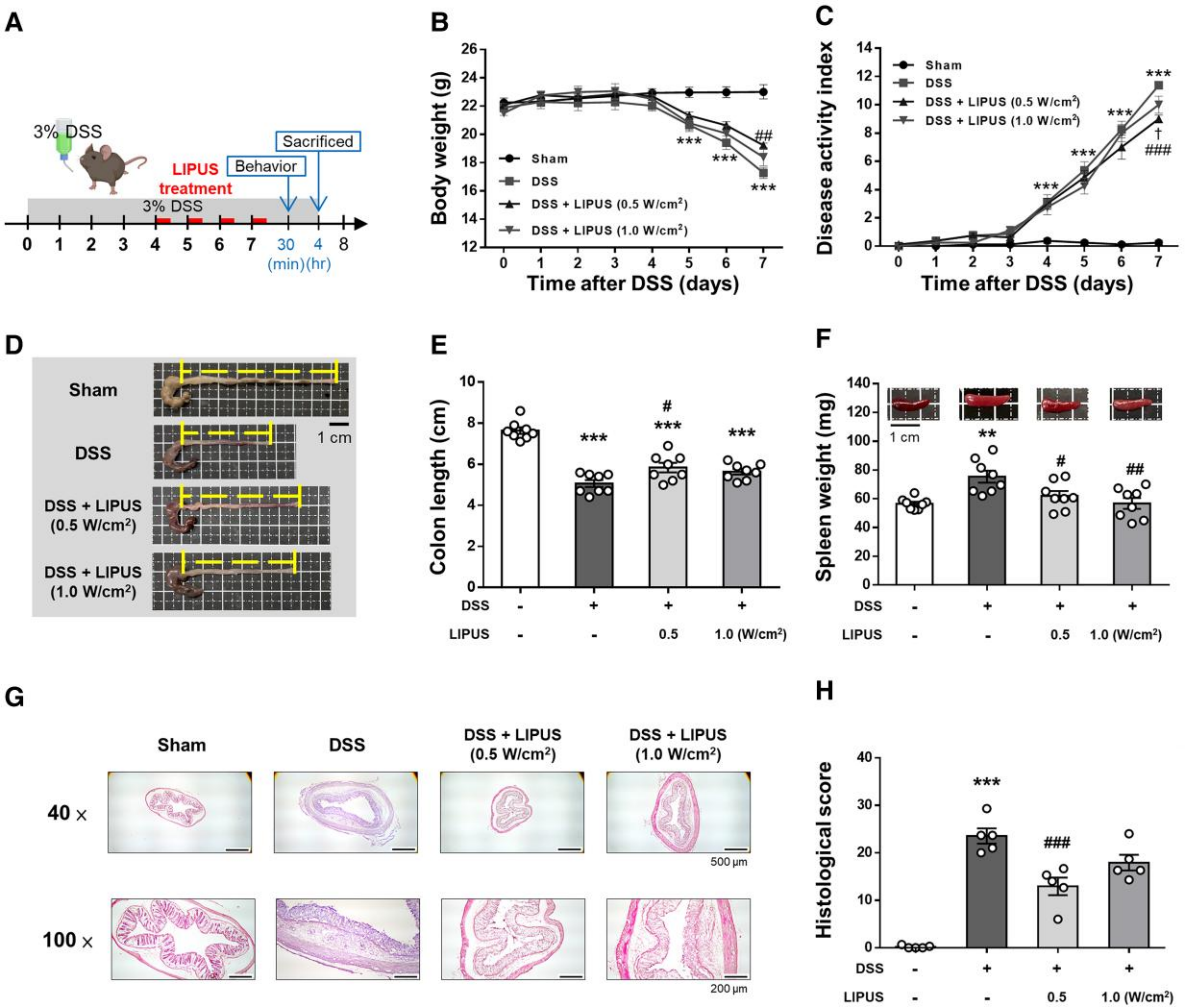
**LIPUS alleviated colon damage in DSS-induced acute colitis.** (**A**) Time schedule of the study. Mice were treated with 3% DSS dissolved in drinking water for 7 days. LIPUS treatment was administered to the brain daily from Days 4 to 7. (**B**) Changes in body weight in each group. (**C**) DAI score of each group (B and C, *n* = 8 for four groups). Representative photographs of colon (**D**) and colonic length measurement (**E**). (**F**) Effect of LIPUS treatment on spleen weight in DSS-induced acute colitis (E and F, *n* = 8 for four groups). Representative images of hematoxylin and eosin staining (**G**) and histological scores (**H**) (H, *n* = 5 for four groups). In (**B**) and (**C**), *^, #,^ and ^†^ denote a significant difference between the DSS group and the Sham group, DSS + LIPUS 0.5 group, and DSS + LIPUS 1.0 group, respectively. In (**E**), (**F**) and (**H**), * and ^#^ denote significant differences from the Sham group and the DSS group, respectively (^#,†^, *P* < 0.05; **^,##^, *P* < 0.01; ***^,###^, *P* < 0.001). All data are shown as mean ± SEM. Dots represent the number of samples (studied animals). One-way ANOVA followed by Tukey’s *post hoc* test. DAI, disease activity index; DSS, dextran sulphate sodium; LIPUS, low-intensity pulsed ultrasound.

The effects of transcranial LIPUS stimulation were evaluated in mice with acute colitis induced by administration of 3% DSS in their drinking water for 7 days. Mice were exposed to LIPUS treatment from Days 4 to 7 (Fig. 2A). Mice in the DSS-only group exhibited the greatest body weight loss (Fig. 2B) and the highest DAI score (Fig. 2C) in comparison to the other groups. Body weight loss in the DSS + LIPUS 0.5 group was significantly reduced at Day 7 compared with that in the DSS group (*P* < 0.01; Fig. 2B). By Day 7, LIPUS therapy led to a notable reduction in the DAI score at both intensities (*P* < 0.05) when compared with the DSS-only group (Fig. 2C). In the DSS-only group, colon length was significantly reduced compared with the Sham group (*P* < 0.001; Fig. 2D and E). However, no significant difference in colon length was observed between the LIPUS-treated groups and the DSS-only group. Additionally, spleen weight in the DSS group was significantly higher than in the Sham group (*P* < 0.01; Fig. 2F). Both LIPUS treatment groups exhibited a significant reduction in spleen weight compared with the DSS group (both *P* < 0.05; Fig. 2F). Pathological changes in colonic tissue were evaluated following H&E staining. Histological scores showed significantly reduced DSS-induced colonic damage with lower crypt destruction and partial epithelial barrier preservation in the DSS + LIPUS 0.5 group compared with the DSS group (*P* < 0.001; Fig. 2G and H).

### Effects of LIPUS on inflammatory factors in mice with DSS-induced colitis

**Figure 3:**
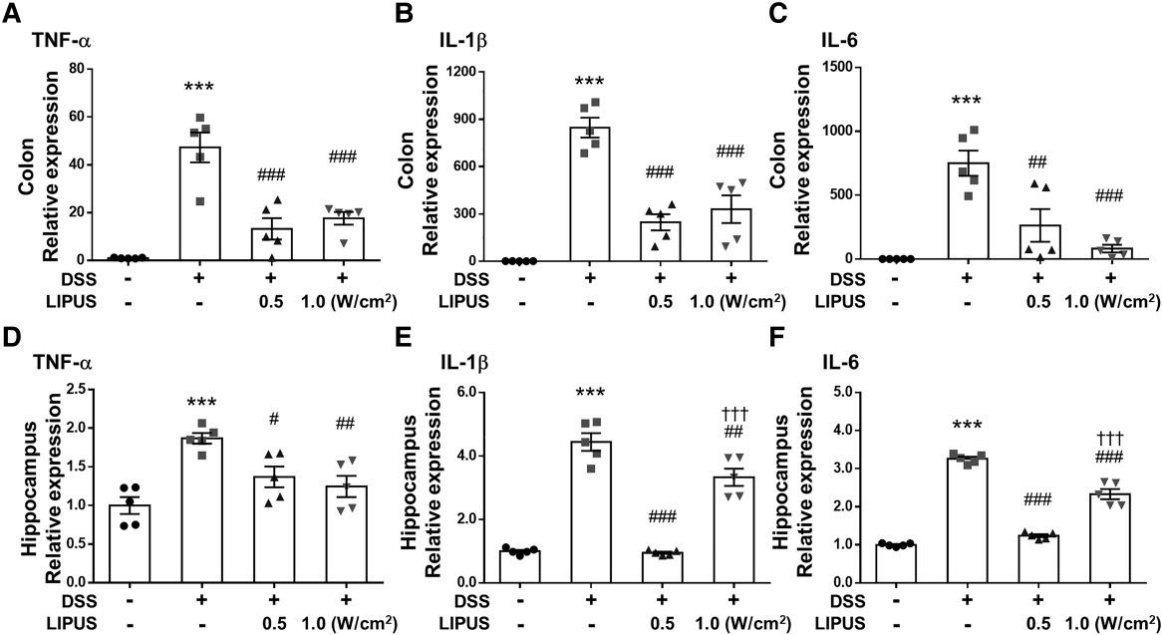
**Effects of LIPUS treatment on inflammatory cytokine expression in mice with DSS-induced colitis.** (**A**) TNF-α, (**B**) IL-1β and (**C**) IL-6 mRNA in colon tissue was quantified with qRT-PCR. (**D**) TNF-α, (**E**) IL-1β and (**F**) IL-6 mRNA in the hippocampus were quantified with qRT-PCR. *^, #,^ and ^†^ denote significant difference from the Sham group, DSS group and DSS + LIPUS 0.5 group, respectively (^#^, *P* < 0.05; ^##^, *P* < 0.01; ***^,###,†††^, *P* < 0.001; *n* = 5). All data are shown as mean ± SEM. Dots represent the number of samples (studied animals). One-way ANOVA followed by Tukey’s *post hoc* test. DSS, dextran sulphate sodium; IL-1β, interleukin-1β; IL-6, interleukin-6; LIPUS, low-intensity pulsed ultrasound; TNF-α, tumor necrosis factor-α.

DSS treatment significantly increased the levels of TNF-α, IL-1β and IL-6 in colonic tissue (all *P* < 0.001; Fig. 3A–C). Mice treated with LIPUS at intensities of 0.5 W/cm² and 1.0 W/cm² exhibited a significant reduction in the levels of TNF-α, IL-1β and IL-6 when compared with the DSS-only treatment group (all *P* < 0.01; Fig. 3A–C). Similarly, DSS treatment significantly increased TNF-α, IL-1β and IL-6 expression in the hippocampus of mice (all *P* < 0.001; Fig. 3D–F). TNF-α, IL-1β and IL-6 expression significantly decreased following LIPUS treatment at both intensities (all *P* < 0.05; Fig. 3D–F).

### LIPUS treatment restored the gut barrier in colitic mice

**Figure 4:**
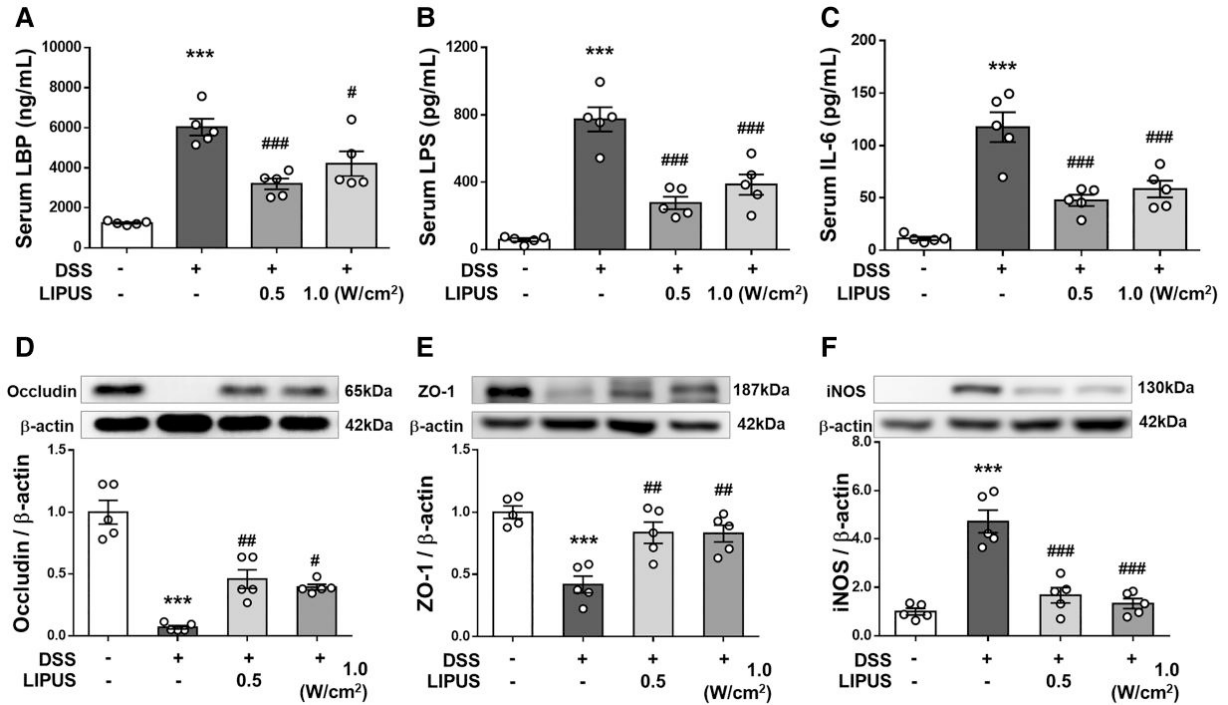
**LIPUS attenuated intestinal barrier defects induced by DSS.** Levels of (**A**) LPB, (**B**) LPS and (**C**) IL-6 in serum. The relative protein levels of (**D**) occludin, (**E**) ZO-1 and (**F**) iNOS in colon tissue. * and ^#^ denote significant differences from the Sham group, DSS group, and DSS + LIPUS 0.5 group, respectively (^#^, *P* < 0.05; ^##^, *P* < 0.01; ***^,###^, *P* < 0.001; *n* = 5). All data are shown as mean ± SEM. Dots represent the number of samples (studied animals). One-way ANOVA followed by Tukey’s *post hoc* test. See supplementary materials for uncropped blots. DSS, dextran sulphate sodium; IL-6, interleukin-6; iNOS, inducible nitric oxide synthase; LBP, LPS-binding protein; LIPUS, low-intensity pulsed ultrasound; LPS, lipopolysaccharide; ZO-1, zonula occludens-1.

Figure 4A–C display the serum levels of LBP, LPS and IL-6 in mice. The integrity of the intestinal barrier could be evaluated by measuring serum concentrations of LBP and LPS. DSS-treated mice showed a marked increase in the levels of LBP and LPS (both *P* < 0.001; Fig. 4A and B), suggesting a higher bacteria-derived antigen burden. However, this increase was significantly diminished after LIPUS treatment at 0.5 W/cm^2^ and 1.0 W/cm^2^ (both *P* < 0.05; Fig. 4A and B). In addition, systemic inflammatory response induced by gut inflammation was indicated by an increase in serum IL-6 (*P* < 0.001; Fig. 4C). Following LIPUS treatment at both intensities, the serum IL-6 level was significantly reduced in comparison to the DSS group (both *P* < 0.001; Fig. 4C). We also examined the effects of LIPUS on major proteins, occludin and ZO-1, involved in the tight junctions of the gut barrier. DSS administration led to a significant decrease in the expression of both occludin and ZO-1 (both *P* < 0.001; Fig. 4D and E). In contrast, occludin and ZO-1 expression significantly increased after LIPUS treatment at both intensities in comparison to the levels observed in the DSS group (*P* < 0.05; Fig. 4D and E). Furthermore, intestinal inflammation was indicated by increased iNOS in the colon (*P* < 0.001; Fig. 4F). LIPUS significantly decreased the iNOS level by 64.6% at 0.5 W/cm^2^ and by 71.8% at 1.0 W/cm^2^ relative to the DSS group (both *P* < 0.001; Fig. 4F).

### Effects of LIPUS on DSS-induced behavioural disorders

**Figure 5:**
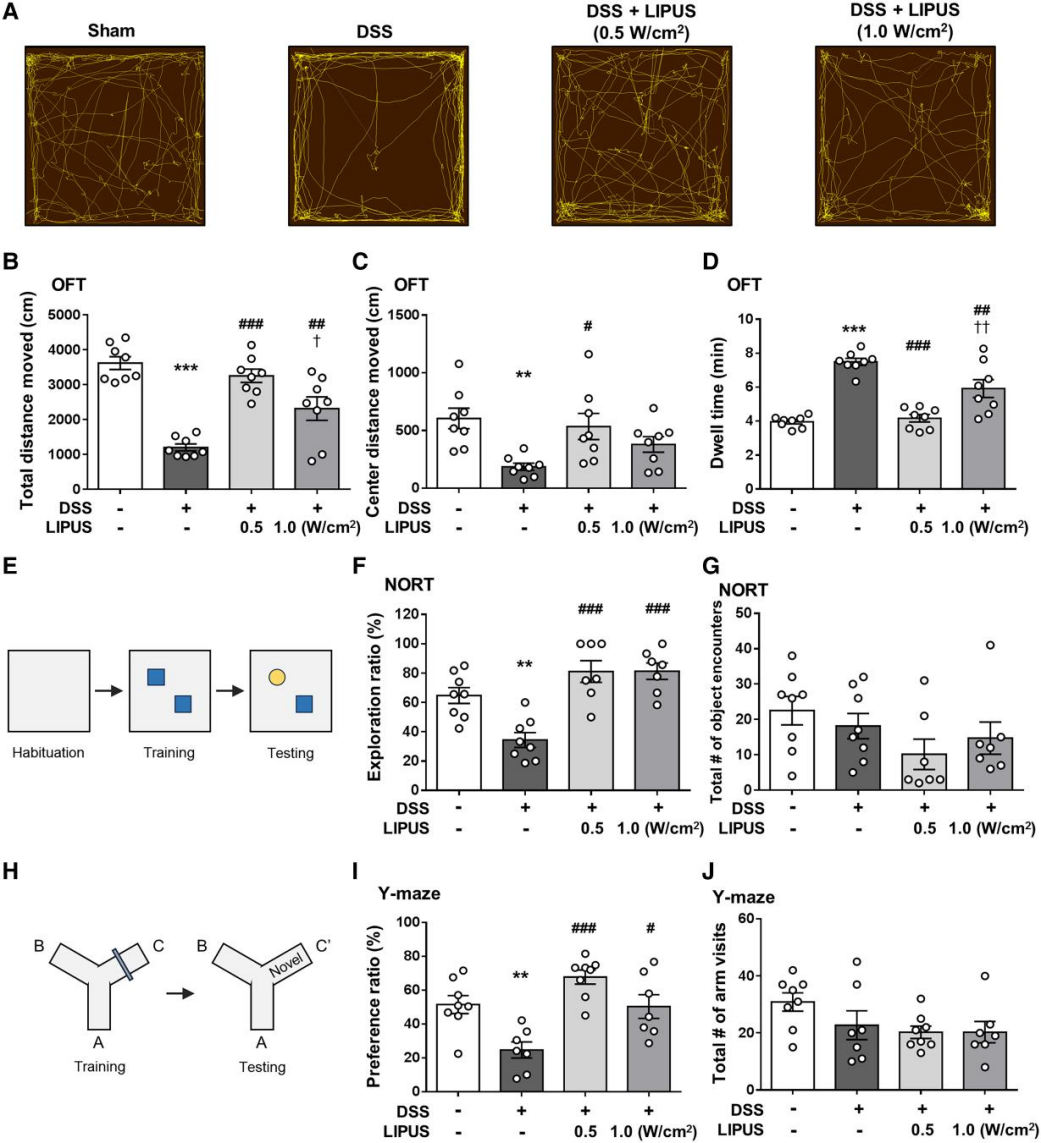
**Effects of LIPUS treatment on DSS-induced behavioural disorders of mice in the behavioural tests.** (**A**) Representative movement tracks in the OFT. (**B**) The distance traveled by mice in the entire OFT area. (**C**) The distance of mice in the central OFT area. (**D**) Result for dwell time (B, C and **D**, *n* = 8 for four groups). (**E**) NORT. (**F**) The exploration ratio of the four groups. (**G**) The number of object encounters of the four groups. A significant object memory impairment was observed in the DSS-treated mice compared with the mice in the Sham group. Both LIPUS treatments attenuated DSS-induced deficits in recognition memory (F and G, *n* = 8 for Sham and DSS groups, *n* = 7 for both LIPUS treatment groups). (**H**) Y-maze test. (**I**) The preference ratio of the four groups. (**J**) The total number of arm visits of the four groups. DSS-treated mice had a lower preference ratio compared with the Sham group. However, these decreases were significantly reversed in DSS plus LIPUS-treated mice (I and J, *n* = 8 for Sham and DSS + LIPUS 0.5 groups, *n* = 7 for DSS and DSS + LIPUS 1.0 groups). *^, #^ and ^†^ denote significant differences from the Sham group, DSS group and DSS + LIPUS 0.5 group, respectively (^#,†^, *P* < 0.05; **^,##,††^, *P* < 0.01; ***^,###^, *P* < 0.001). All data are shown as mean ± SEM. Dots represent the number of samples (studied animals). One-way ANOVA followed by Tukey’s *post hoc* test. DSS, dextran sulphate sodium; LIPUS, low-intensity pulsed ultrasound; NORT, novel object recognition test; OFT, open field test.

The OFT has often been used in the assessment of depression and anxiety-like behaviours. The spontaneous movement of mice in OFT was shown in Fig. 5A. The total distances moved in the entire and central OFT areas by the mice in the DSS group were significantly lower than the movement distances of mice in the Sham group However, mice in both LIPUS intensity groups demonstrated a substantial increase in the total distance traveled throughout the area, in contrast to those in the DSS group (both *P* < 0.01; Fig. 5B). At 0.5 W/cm^2^, LIPUS significantly increased the total distance moved across the central area compared with the DSS group (*P* < 0.05; Fig. 5C). The DSS-treated mice showed depressive and anxiety-like behaviours (Fig. 5D), demonstrated as a longer dwell time (*P* < 0.001). In comparison with the DSS group, LIPUS therapy at both power levels notably improved this behavioural impairment (*P* < 0.01; Fig. 5D).

The NOR task was used to assess recognition memory (Fig. 5E). Compared with the Sham group, DSS-treated mice revealed impairment in recognition memory as indicated by a significantly lower exploration ratio (*P* < 0.01; Fig. 5F). Both intensities of LIPUS treatment significantly improved deficits in recognition memory following induction of colitis in comparison to the DSS group (both *P* < 0.001; Fig. 5F). The total number of encounters with object was quantified, with no significant difference between the four groups (Fig. 5G). Additionally, the Y-maze test was used to assess spatial recognition memory (Fig. 5H). Mice treated with DSS displayed deficits in spatial recognition memory, demonstrated by a notably reduced preference ratio (*P* < 0.01; Fig. 5I). LIPUS therapy at both intensities markedly enhanced spatial memory impairments relative to the DSS-only group (both *P* < 0.05; Fig. 5I). The overall number of arm entries was similar among the four groups, indicating that neither DSS administration nor the combination of DSS and LIPUS treatment had an impact on locomotor activity (Fig. 5J).

### Effects of LIPUS on the intestinal microbial structure in mice with DSS-induced colitis

**Figure 6:**
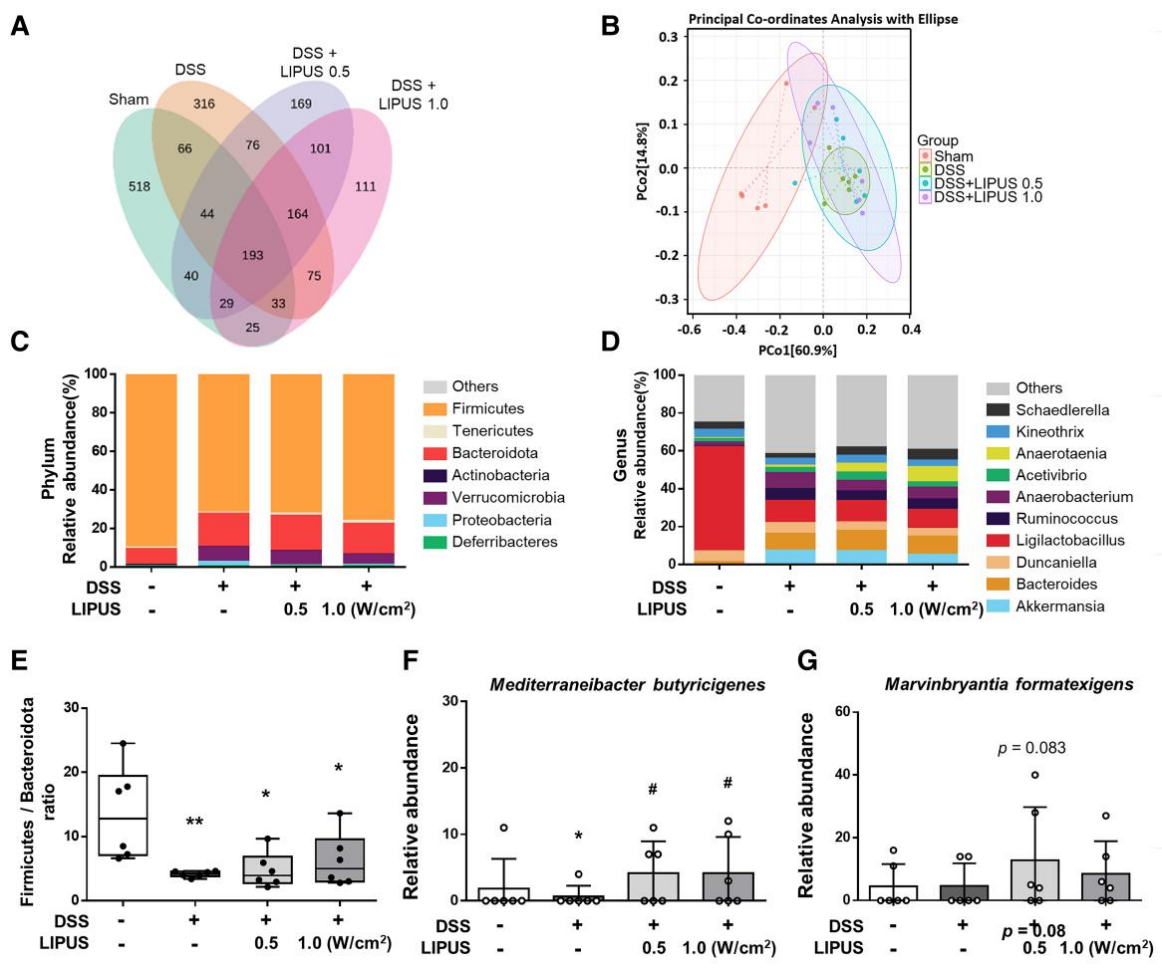
**Effects of LIPUS treatment on the gut microbiome structure in mice with DSS-induced colitis.** (**A**) Venn diagrams of OUTs showing overlap between groups. (**B**) PCoA was based on Bray–Curtis distances (B, *n* = 6 for four groups). (**C**) Relative abundance at the phylum level of each group. (**D**) Relative abundance at the genus level of each group. (**E**) The ratio of *Firmicutes* to *Bacteroidota* of different groups at the phylum level. (**F**) The level of *M. butyricigenes*. (**G**) The level of *M. formatexigens* (E, F and G, *n* = 6 for four groups). All data are shown as mean ± SEM. Dots represent the number of samples (studied animals). One-way ANOVA followed by Tukey’s *post hoc* test. *^, #^ and ^†^ denote significant differences from the Sham group, DSS group and DSS + LIPUS 0.5 group, respectively (*^,#^, *P* < 0.05; **, *P* < 0.01). DSS, dextran sulphate sodium; LIPUS, low-intensity pulsed ultrasound.

The V1–V9 region of the 16S rDNA gene in the intestinal contents of mice was sequenced. Cluster analysis was performed for the Sham, DSS, DSS combined with LIPUS at 0.5 W/cm², and DSS combined with LIPUS at 1.0 W/cm² groups. The number of unique OUT values in each group was higher than 111, and the number of common OUT values in all groups was 193, as shown in Fig. 6A. β-diversity was visualized using PCoA to detect differences in the microbial community between multiple samples (Fig. 6B). The distance between groups reflected the degree of difference. The results showed that there was a certain distance between the Sham and the DSS group. However, unlike the DSS group, both LIPUS groups were relatively similar to the Sham group. The composition of the microbiota at the level of phylum is shown in Fig. 6C. In the Sham group, the most prevalent bacterial phyla were *Firmicutes* (89.95%) and *Bacteroidota* (8.24%). DSS administration notably influenced the proportional abundances of these taxa; it reduced the relative abundance of *Firmicutes* to 71.74% and increased those of *Bacteroidota* and *Verrucomicrobia* to 17.33% and 7.33%, respectively. The relative abundances of the taxa in the DSS group and both LIPUS treatment groups did not differ, except for the reduced relative abundance of *Proteobacteria* in the LIPUS groups. At the genus level, DSS caused a significant decrease in the abundance of the genus *Ligilactobacillus* (Fig. 6D), while increasing the abundances of *Akkermansia*, *Bacteroides*, and *Anaerobacterium*. LIPUS treatment reduced the relative abundance of *Anaerobacterium* and increased those of *Anaerotaenia* and *Schaedlerella*. A reduced ratio of *Firmicutes* to *Bacteroidota* (F/B) is linked to IBD.^34^ In contrast to the Sham group, in the DSS-treated mice, there was a notable reduction in the F/B ratio (*P* < 0.01; Fig. 6E). In both LIPUS treatment groups, an increasing, but non-significant, trend was observed in the F/B ratio, and a more in-depth analysis identified clear differences between the bacterial groups at the genus level. The relative abundance of *Mediterraneibacter butyricigenes* showed a marked decrease in DSS group (*P* < 0.05; Fig. 6F). The proportion of the species *M. butyricigenes* significantly increased in both LIPUS treatment groups compared with that in the DSS group (both *P* < 0.05; Fig. 6F). Furthermore, there was a non-significant trend toward an increase in the proportion of *Marvinbryantia formatexigens* in both LIPUS-treated groups relative to the DSS group (Fig. 6G).

## Discussion

Neuroinflammation can be triggered by inflammation in the colon. In this study, we provide new information on the impact of transcranial LIPUS treatment on the outcomes of neuroinflammation and colonic inflammation induced by DSS. This study investigated the effects of transcranial LIPUS, as a potential non-invasive treatment for DSS-induced colitis, via inhibition of neuroinflammation. Our results demonstrated that transcranial LIPUS not only reduced proinflammatory cytokine levels in the hippocampus but also alleviated inflammation in the colon. Furthermore, LIPUS improved behavioural abnormalities, mitigated intestinal barrier dysfunction, and decreased serum levels of LPS and LBP. However, transcranial LIPUS did not obviously alter the composition of the gut microbiota except for the microbial community differences in DSS-induced colitis.

The nervous system and the gastrointestinal tract communicate through a bidirectional network of pathways, called the gut–brain axis, that includes neural, endocrinal, immune, and humoral links.^35^ Although IBD is increasingly linked to neuroinflammation and behavioural dysfunctions, the connection between colonic inflammation and neuroinflammation remains unknown. LIPUS is used in non-invasive physical therapy and has the ability to induce biological effects through mechanotransductive effects.^36^ Transcranial LIPUS has been shown to attenuate neuroinflammation and improve memory impairments in mice systemically administered with LPS.^25^ In order to explore the influence of anti-neuroinflammation on IBD, LIPUS brain stimulation was applied to the brain with inflammation caused by DSS-induced colitis. The results showed that transcranial LIPUS treatment restored the gut barrier function, reduced proinflammatory cytokines in the brain and colon, and downregulated LPB and LPS in the serum (Figs 2G, 3 and 4). These findings support the hypothesis that LIPUS can alleviate colitis by inhibiting inflammation of the brain without direct treatment of the intestine. This study demonstrated that anti-neuroinflammation caused by transcranial LIPUS can feed back and change gut function and recovery after IBD via the brain-to-gut signaling axis.

Consistent with previous reports, we found that colitis alters the gut microbiota composition and increases the concentrations of proinflammatory substances.^15,37^ The relative proportions of key bacterial phyla are commonly recognized as playing a crucial role in shaping the composition of the gut microbiota.^38^ The decrease in the F/B ratio is regarded as an indication of IBD. To the best of our knowledge, this is the first study to examine the effect of transcranial LIPUS therapy on the gut microbiota in the setting of DSS-induced colitis. In general, our data revealed that the composition of the gut microbiota and the F/B ratio did not change after transcranial LIPUS stimulation, which, to the best of our knowledge, has not been reported before. However, the relative abundances of *M. butyricigenes* and *M. formatexigens* increased in both LIPUS treatments following DSS administration (Fig. 6F and G). The increased relative abundance of *Mediterraneibacter* can enhance butyrate production in the intestine, thereby improving inflammation.^39^  *Marvinbryantia formatexigens* may help to improve fermentation and produce acetate and lactate via glucose fermentation.^40,41^ In addition, the microbial community in both LIPUS treatment groups was relatively similar to that in the Sham group but different from that in the DSS-only group (Fig. 6B).

Depression, anxiety and memory impairments are associated with intestinal inflammation.^42^ DSS-induced colitis triggers an inflammatory response in the brain that is accompanied by anxiety-like behaviour and impaired recognition memory.^43^ Our results were in line with previous findings that DSS-induced colitis led to neuroinflammation, as evidenced by an increase in the production of TNF-α, IL-1β and IL-6 in the brain (Fig. 3D and E). Recently, evidence has emerged for the gut microbiota having a role in mediating brain disorders.^32,37^ Using 16S sequencing to study the overall composition of the microbiota, we also demonstrated that DSS-induced colitis led to significant changes in the microbiota (Fig. 6). Despite transcranial LIPUS treatment being able to attenuate DSS-induced neuroinflammation, the disrupted composition of the microbiota cannot be improved by LIPUS. In this study, behavioural assessments showed anxiety-like and memory impairment symptoms in mice with DSS-induced colitis, while LIPUS treatment helped reduce the behavioural disturbances caused by DSS (Fig. 5). Therefore, our findings suggest that transcranial LIPUS treatment may modulate behaviour, including anxiety-like behaviour and impaired recognition memory, partly by attenuation of inflammation in the brain and gut.

The inflammatory response that occurs during tissue injury has beneficial aspects and may help to remove toxic agents and inhibit their detrimental effects. However, this inflammation may potentiate secondary tissue injury.^44^ Many therapies inhibit the inflammatory response in IBD, but there is no cure for the disease.^45,46^ The reason may be that systemic therapies with drugs prevent much of the beneficial aspects of this inflammatory response. The development of a more targeted treatment may be the key direction for effective therapy. Thus, local and targeted LIPUS stimulation on specific organs has the potential to be an effective approach to the improvement of outcomes of patients with IBD and neuroinflammation. Our results showed that local LIPUS treatment reduced the severity of colitis, most likely through stimulation of the brain, leading to attenuation of systemic inflammation. Therefore, we speculate that the brain–gut axis may contribute to the beneficial effects of transcranial LIPUS in reducing DSS-induced symptoms of colitis. Further investigations should focus on understanding the signals that cause the communication between the brains and gut so LIPUS stimulation can serve as a targeted local treatment for neuroinflammation and IBD via local anti-inflammatory effects.

This study demonstrated that colitis causes inflammation in the brain and increased inflammatory cytokines in the serum and that neuroinflammation and colonic inflammation were alleviated by transcranial LIPUS treatment. Our findings revealed that the gut–brain axis was likely to play an important role in the protective effect of LIPUS against behavioural disorders and the symptoms of colitis induced by DSS. Future investigations should focus on the connection between the brain and gut to gain further insights into the therapeutic mechanisms of transcranial LIPUS. Finally, our results open novel avenues for using physical ultrasound stimulation to investigate treatments for brain disorders and the source of inflammation.

## Supplementary Material

fcaf119_Supplementary_Data

## Data Availability

The datasets used and/or analysed during the current study are available from the corresponding author upon reasonable request.
